# Beyond the numbers: understanding inter-center variation in recanalization therapy rates

**DOI:** 10.3389/fneur.2026.1898540

**Published:** 2026-10-26

**Authors:** Yousef Quzeih, Anastassia Kolde, Riina Vibo, Teele Tuularu, Katrin Sikk, Janika Kõrv

**Affiliations:** 1 Faculty of Medicine, University of Tartu, Tartu, Estonia; 2 Institute of Mathematics and Statistics, University of Tartu, Tartu, Estonia; 3 Institute of Genomics, University of Tartu, Tartu, Estonia; 4 Department of Neurology, Tartu University Hospital, Tartu, Estonia; 5 Department of Neurology, North Estonia Medical Centre, Tallinn, Estonia

**Keywords:** mild stroke, recanalization therapy, stroke, stroke care, thrombolysis

## Abstract

**Introduction:**

This study aimed to investigate the inter-center differences in recanalization therapy (RT) utilization between Estonia’s two largest comprehensive stroke centers and to assess whether variation in the management of patient subgroups is associated with differences in short-term neurological and functional outcomes.

**Methods:**

We performed a retrospective two-center study including all acute ischemic stroke (AIS) patients admitted between January–June 2021. Mild AIS or mild stroke was defined as National Institutes of Health Stroke Scale (NIHSS) ≤ 5. Demographic, treatment, NIHSS, and modified Rankin Scale (mRS) data were analyzed using nonparametric and categorical tests. Multivariable logistic regression evaluated predictors of RT use in AIS and predictors of stroke outcome.

**Results:**

Among 635 AIS patients, RT was administered more frequently at Tartu University Hospital (TUH) than at North Estonia Medical Centre (NEMC) (45.2% vs. 30.7%, *p* < 0.001; aOR 0.49, 95% CI 0.33–0.74). A total of 301 (47.5%) patients had mild stroke. RT use in mild stroke was substantially higher at TUH (33.1% vs. 10.0%, *p* < 0.001). Descriptive analysis showed greater neurological improvement in RT-treated mild stroke patients than non-RT-treated patients [median NIHSS reduction 2.0 (IQR 0.8–3.0) vs. 1.0 (IQR 0.0–2.0), respectively; Wilcoxon rank-sum test, *p* < 0.001], whereas good functional outcome did not differ by RT exposure. After adjustment, neurological improvement was independently associated with treatment center and baseline NIHSS category but not with RT exposure (aOR 0.94, 95% CI 0.43–2.06).

**Discussion:**

RT was also not independently associated with good functional outcome. Inter-center variation in RT utilization was primarily driven by the management of mild stroke patients. Comparable stroke-care infrastructure did not ensure uniform RT utilization.

## Introduction

Timely intravenous thrombolysis (IVT) and mechanical thrombectomy (MT) are pivotal recanalization therapies (RT) for reducing brain injury and improving outcomes in acute ischemic stroke (AIS) (1, 2). Across Europe, substantial efforts have been made to optimize access to and delivery of RT through European Stroke Organization (ESO) guidelines, national quality initiatives, stroke unit certification, and performance-driven programs such as the Stroke Action Plan for Europe (SAP-E). Despite this, marked variation in RT utilization persists between and within countries, indicating that adherence to guidelines alone does not fully explain differences in performance indicators. Determinants of such variation remain incompletely understood, particularly in healthcare systems where structural resources and guidelines are similar.

Estonia has two comprehensive stroke centers that now hold ESO certification (3), Tartu University Hospital (TUH) and North Estonia Medical Centre (NEMC), which serve comparable populations under the same national and European treatment framework. Nevertheless, national quality indicators have reported higher RT rates at TUH than at NEMC since 2019 (4). In such settings with minimal structural, demographic, and organizational differences, variation in RT utilization may reflect differences in patient selection or treatment patterns rather than infrastructure alone. This may be particularly relevant for patient subgroups with less clearly defined treatment indications.

The primary aim of this study was to investigate the inter-center differences in RT utilization between Estonia’s two largest comprehensive stroke centers and to assess whether variation in the management of patient subgroups is associated with differences in short-term neurological and functional outcomes.

## Materials and methods

This retrospective observational study included all consecutive AIS patients admitted to TUH (*n* = 332) and NEMC (*n* = 303) between January 1 and June 30, 2021. The catchment areas for acute stroke for TUH and NEMC are around 300,000 and 500,000, respectively. Both centers function as comprehensive stroke centers within a centralized national stroke care system with standardized EMS triage pathways. Patients were managed in stroke units by a multidisciplinary team according to European and international guidelines and institutional protocols with no substantial differences between the hospitals. In both centers, non-disabling neurological deficits were considered a contraindication to reperfusion treatment, and treatment decisions were not based solely on the total NIHSS score.

RT included IVT, MT, or both. IVT and MT were available within extended time windows, with CT perfusion available for wake-up strokes 24/7 at both centers. RT non-recipients were managed according to best medical therapy.

Data for patients who received RT (RT recipients) were retrieved from the prospectively collected SITS registry (Safe Implementation of Treatment in Stroke). The patients who did not receive RT (RT non-recipients) were identified from the hospitals’ electronic databases using the ICD-10 codes I63.0–I63.9. Collected variables included age, sex, NIHSS score on admission and at discharge, modified Rankin Scale (mRS) score before stroke and at discharge. Mild AIS (mild stroke) was defined as NIHSS score ≤5. Onset-to-door time (ODT) was calculated using arrival times and reported onset time.

The primary outcome was neurological improvement, and the secondary outcome was functional outcome. Neurological improvement was defined as a decrease of ≥1 point in NIHSS score between admission and discharge. Functional outcome was assessed relative to premorbid functional status; good functional outcome was defined as discharge mRS equal to premorbid mRS.

Continuous variables were summarized as median (interquartile range, IQR) and compared using the Wilcoxon rank-sum test. Categorical variables were summarized as counts and percentages and compared using the Chi-squared test or Fisher’s exact test as appropriate. Yates’ continuity correction was applied for 2 × 2 tables.

Multivariable logistic regression models were fitted among all AIS patients to assess predictors of RT utilization, and separately among mild stroke patients to assess predictors of neurological improvement and good functional outcome, as defined above. Sensitivity analysis excluded treatment centers to assess the stability of associations given the correlation between treatment center and RT utilization. The RT utilization model included treating hospital, stroke severity (mild stroke vs. severe), age, sex, and standard IVT treatment window eligibility (ODT ≤ 4.5 h) as covariates. In analyses restricted to mild stroke patients, models additionally included baseline NIHSS category (0–2 vs. 3–5) to account for initial stroke severity. In IVT-focused sensitivity analyses, the mild-stroke analyses were repeated after excluding patients treated with MT without IVT, while patients receiving both IVT and MT were retained in the IVT group. For the purposes of modelling, arrival beyond 4.5 h was classified as late arrival.

To assess the robustness of the primary neurological outcome to potential variability associated with a one-point NIHSS change, a sensitivity analysis was performed using a more stringent definition of neurological improvement (≥2-point decrease in NIHSS between admission and discharge).

Missing data were assessed separately for each variable. No values were imputed, and patients with missing data were excluded only from analyses requiring the relevant variable. Multicollinearity was assessed using variance inflation factors (VIFs), and model calibration was evaluated using the Hosmer–Lemeshow goodness-of-fit test. Two-sided *p*-values < 0.05 were considered statistically significant. All analyses were performed in R (version 4.5.1).

This study was approved by the University of Tartu Human Research Ethics Committee (license No. 369/T-6, 17 October 2022). The requirement for informed consent was waived due to the retrospective design and use of pseudoanonymized data. All procedures were conducted in accordance with the Declaration of Helsinki and relevant national regulations.

## Results

### All AIS patients

During the study period, a total of 635 patients with acute ischemic stroke (AIS) were admitted to the two centers and included in the final analysis (Figure 1). Baseline demographic characteristics, ODT ≤ 4.5 h, and stroke severity distributions of all AIS patients were similar between TUH and NEMC (Supplementary Table 1). Nevertheless, RT was administered at a significantly higher rate in TUH than in NEMC (45.2% vs. 30.7%; *p* < 0.001).

**Figure 1:**
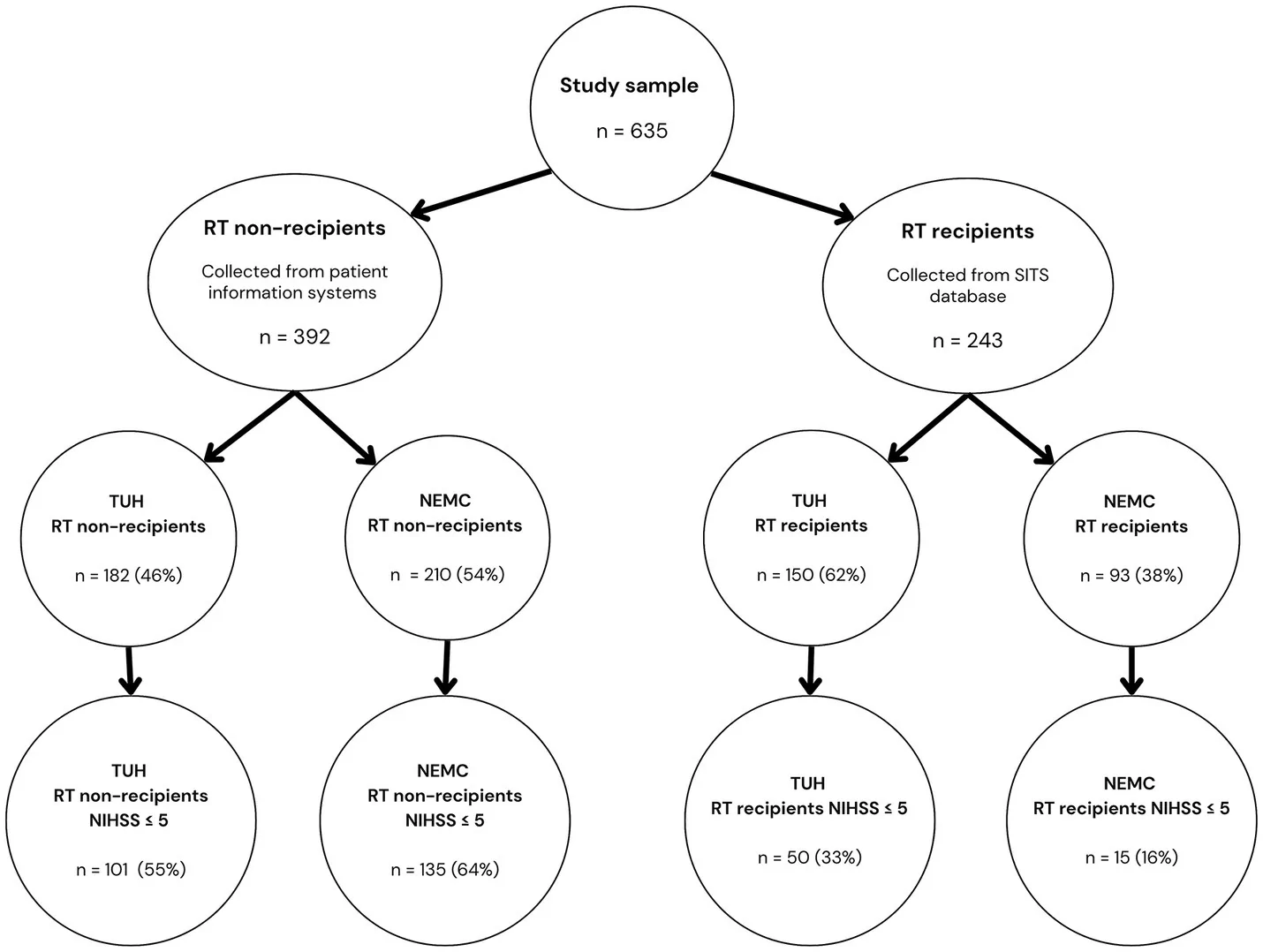
Flowchart of patient inclusion and study population selection. The study cohort included 635 patients with acute ischemic stroke. Patients were categorized according to receipt of recanalization therapy and treatment center. Subgroups with mild stroke (NIHSS ≤5) are shown.

When AIS patients were stratified by RT status (Supplementary Table 2), TUH RT recipients had lower admission NIHSS scores than NEMC RT recipients [median 8 (IQR 4.0–15.8) vs. 12.0 (IQR 6.0–19.0); *p* = 0.030]. Other between-center differences within treatment strata were small and not statistically significant.

A multivariable logistic regression model was performed to identify predictors of recanalization therapy (RT) use among all acute ischemic stroke patients. Treatment at NEMC was independently associated with lower odds of receiving RT compared with TUH. Severe stroke presentation and earlier arrival were strong predictors of RT administration, while increasing age showed a negative association. Sex was not independently associated with RT use (Table 1).

**Table 1:** Multivariable logistic regression analysis of predictors of recanalization therapy (RT) utilization among all acute ischemic stroke (AIS) patients.

Predictor of RT utilization	aOR	95% CI	*p*-value
Treatment center (NEMC vs. TUH)	0.49	0.33–0.74	<0.001
Stroke severity (NIHSS >5 vs. ≤ 5)	4.85	3.12–7.54	<0.001
Late arrival (>4.5 h vs. ≤ 4.5 h)	0.09	0.06–0.13	<0.001
Age (per year increase)	0.98	0.96–0.99	0.006
Sex (male vs. female)	1.08	0.70–1.67	0.72

Values represent aOR with 95% confidence intervals from multivariable logistic regression. Reference categories were TUH for treatment center, NIHSS ≤5 for stroke severity, onset-to-door time ≤4.5 h for arrival time, and female sex. RT includes intravenous thrombolysis, mechanical thrombectomy, or both. AIS, acute ischemic stroke; RT, recanalization therapy; TUH, Tartu University Hospital; NEMC, North Estonia Medical Centre; NIHSS, National Institutes of Health Stroke Scale.

Notably, mild stroke patients were significantly more likely to receive RT in TUH (Table 1). When patients with mild stroke were excluded, RT rates between centers were similar (TUH 55.2% vs. NEMC 51.0%; *p* = 0.51). These findings supported further subgroup analysis focused on this population.

### Mild stroke patients

A total of 301 (47.5%) patients had mild stroke: 151/331 at TUH (45.6%) and 150/303 at NEMC (49.5%; Table 2). Mild stroke patients were stratified according to treatment center and RT status. Baseline characteristics and arrival times were generally comparable between centers, although pre-stroke mRS differed significantly between TUH and NEMC. Despite this, RT was administered to 33.1% of mild stroke patients at TUH compared with 10.0% at NEMC (*p* < 0.001), corresponding to approximately a threefold difference in crude treatment rates. An IVT-focused sensitivity analysis excluding the five MT-only recipients similarly demonstrated higher IVT utilization at TUH than at NEMC (32.7% vs. 7.5%, *p* < 0.001).

**Table 2:** Baseline characteristics and outcomes of patients with mild acute ischemic stroke (mild stroke) (*n* = 301) stratified according to treatment center and recanalization therapy status.

Variable	Mild stroke patients					
	Treatment center			Treatment status		
	TUH (*n* = 151)	NEMC (*n* = 150)	*p*-value	RT recipients (*n* = 65)	RT non-recipients (*n* = 236)	*p*-value
Age, median (IQR)	73.0 (64.0–81.0)	73.0 (61.2–80.0)	0.398	68.0 (62.0–76.0)	74.0 (64.0–81.0)	0.009
Male sex, *n* (%)	80 (53.0)	84 (56.0)	0.682	39 (60)	125 (53.0)	0.386
Early arrival, *n* (%)	49 (32.5)	36 (24.0)	0.134	48 (73.8)	37 (15.7)	<0.001
Late arrival, *n* (%)	102 (67.5)	114 (76.0)		17 (26.2)	199 (84.3)	
NIHSS 0–2, arrival, *n* (%)	67 (44.4)	74 (49.3)	0.455	17 (26.2)	124 (52.5)	<0.001
NIHSS 3–5, arrival, *n* (%)	84 (55.6)	76 (50.7)		48 (73.8)	112 (47.5)	
NIHSS on discharge, median (IQR)	1.0 (0.0–2.8)	2.0 (0.0–3.0)	0.024	1.0 (0.0–3.0)	1.0 (0.0–3.0)	0.065
NIHSS improvement, median (IQR)	1.0 (0.0–2.0)	0.0 (0.0–2.0)	<0.001	2.0 (0.8–3.0)	1.0 (0.0–2.0)	<0.001
Pre-stroke mRS 0–1, *n*/*N* (%)	112/151 (74.2)	126/134 (94.0)	<0.001	56/65 (86.2)	182/220 (82.7)	0.643
Pre-stroke mRS ≥ 2, *n*/*N* (%)	39/151 (25.8)	8/134 (6.0)		9/65 (13.8)	38/220 (17.3)	
Good functional outcome, *n*/*N* (%)	69/151 (45.7)	38/134 (28.4)	0.004	27/65 (41.5)	80/220 (36.4)	0.541

Values are presented as median (interquartile range) or number (%). Mild stroke was defined as NIHSS ≤5 at admission. RT includes intravenous thrombolysis, mechanical thrombectomy, or both. Neurological improvement was defined as a decrease of ≥1 point in NIHSS between admission and discharge. Good functional outcome was defined as discharge mRS equal to premorbid mRS. Early arrival was defined as onset-to-door time ≤4.5 h and late arrival as >4.5 h. *p* values are reported for the overall comparison of each binary variable and are therefore shown only once for complementary categories. TUH, Tartu University Hospital; NEMC, North Estonia Medical Centre; NIHSS, National Institutes of Health Stroke Scale; mRS, modified Rankin Scale; RT, recanalization therapy.

When stratified by treatment status, RT-treated mild stroke patients were younger, arrived earlier, and had higher NIHSS scores on admission (Table 2). A total of 10 patients with mild stroke underwent MT (5 at each center), including 5 who received combined IVT and MT; only one such case occurred at NEMC.

### Outcomes of mild stroke patients

Neurological and functional outcomes among mild stroke patients were first assessed using changes in NIHSS and mRS scores between admission and discharge. NIHSS scores at discharge were significantly lower in TUH (Table 2). Correspondingly, neurological recovery measured by reduction in NIHSS scores was greater among TUH mild stroke patients compared with those treated at NEMC (Table 2).

When stratified by treatment status, RT-treated mild stroke patients demonstrated greater neurological improvement. Good functional outcome was more frequent at TUH than at NEMC. When stratified by treatment status, good functional outcome did not differ significantly between RT recipients and non-recipients (Table 2).

### Multivariable outcome analyses of mild stroke patients

In a multivariable logistic regression model assessing predictors of neurological improvement among mild stroke patients, treatment at TUH and baseline NIHSS group 3–5 were the strongest predictors of neurological improvement. Increasing age showed a modest negative association. Receiving RT was not independently associated with neurological improvement in multivariable models (Table 3).

**Table 3:** Multivariable logistic regression analysis of predictors of neurological improvement and good functional outcome among patients with mild acute ischemic stroke (mild stroke).

Predictor	NIHSS improvement			Good functional outcome		
	aOR	95% CI	*p*-value	aOR	95% CI	*p*-value
Treatment center: NEMC vs. TUH	0.38	0.23–0.65	<0.001	0.4	0.26–0.75	0.003
Baseline NIHSS 3–5 vs. 0–2	4.30	2.56–7.23	<0.001	0.5	0.32–0.91	0.020
Age (per year)	0.98	0.96–1.00	0.043	1.0	0.97–1.01	0.146
RT use (yes vs. no)	0.94	0.43–2.06	0.880	0.7	0.35–1.54	0.409
Sex (male vs. female)	0.96	0.57–1.63	0.880	1.4	0.82–2.31	0.233
Onset-to-door (>4.5 h vs. ≤4.5 h)	0.77	0.40–1.49	0.440	1.4	0.73–2.72	0.313

Adjusted odds ratios (aOR) with 95% confidence intervals were obtained from multivariable logistic regression including treatment center, baseline NIHSS category (0–2 vs. 3–5), age, sex, onset-to-door time ≤4.5 h, and recanalization therapy use. Neurological improvement was defined as a decrease of ≥1 point in NIHSS between admission and discharge. Good functional outcome was defined as discharge mRS equal to premorbid mRS. TUH, Tartu University Hospital; NEMC, North Estonia Medical Centre; NIHSS, National Institutes of Health Stroke Scale; RT, recanalization therapy.

Because the treatment center was strongly associated with RT utilization, a sensitivity analysis was performed using a model that excluded the treatment-center variable. This analysis yielded similar results, indicating that the observed associations were not driven solely by inter-center differences (Supplementary Table 3). A separate sensitivity analysis using the more stringent neurological outcome (≥2-point decrease in NIHSS) yielded results consistent with the primary analysis (Supplementary Table 4).

In a separate multivariable model evaluating functional outcome, treatment at TUH and lower baseline NIHSS severity were independently associated with higher odds of good functional outcome (Table 3).

IVT-focused sensitivity analyses excluding the five MT-only recipients yielded similar results, and the associations with the treatment center remained unchanged. Compared with IVT recipients, IVT non-recipients did not have significantly different odds of neurological improvement (aOR 0.82, 95% CI 0.35–1.92; *p* = 0.640) or good functional outcome (aOR 0.65, 95% CI 0.29–1.44; *p* = 0.291).

No evidence of problematic multicollinearity was observed in the multivariable models (all VIFs <1.7). Hosmer–Lemeshow tests indicated no evidence of lack of fit for the RT-utilization (*p* = 0.143), neurological-improvement (*p* = 0.242), or functional-outcome (*p* = 0.436) models.

## Discussion

This study found that variation in RT utilization between two comprehensive stroke centers within the same national healthcare system was primarily observed in mild stroke patients. Despite comparable patient characteristics, RT was more frequently administered in mild stroke patients at TUH. RT-treated mild stroke patients demonstrated greater early neurological improvement in descriptive analyses; however, this association was not retained after adjustment in multivariable models. In descriptive analyses, good functional outcome was more frequent at TUH than at NEMC among all mild stroke patients, whereas no between-center difference was observed among RT-treated mild stroke patients, suggesting that the observed center-level differences in short-term outcomes cannot be attributed to RT utilization alone. Despite the different eligibility criteria for IVT and MT, IVT-focused sensitivity analyses yielded findings consistent with the primary analyses.

Previous studies have demonstrated substantial variation in the treatment of mild stroke. Willey et al. (5) reported that the proportion of IVT-treated patients with NIHSS 0–3 ranged from 2.7 to 18.0% across a multicenter stroke network, reflecting ongoing uncertainty regarding treatment of the mildest strokes. More recent registry data likewise show that mild symptoms remain a common reason for withholding IVT despite increasing treatment rates over time (6). Our findings suggest that such variation may persist even between structurally comparable comprehensive stroke centers operating within the same national healthcare system.

In multivariable models, neurological improvement was independently associated with the baseline NIHSS category (3–5) and treatment at TUH, rather than RT exposure itself. Furthermore, increasing age showed a modest negative association. These findings suggest that differences in patient selection and treatment thresholds between centers may contribute to the observed differences in short-term neurological recovery among mild stroke patients.

Differences in baseline patient characteristics further support this interpretation. RT-treated mild stroke patients were younger, arrived earlier, and had slightly higher baseline NIHSS scores, all known factors to influence post-stroke neurological recovery. Taken together, this study provides real-world evidence from centers within the same national healthcare system, allowing assessment of variation that is less confounded by structural differences than in prior multi-national studies.

Within the ESO SAP-E, IVT and MT utilization are monitored as key performance indicators of stroke care delivery (7). In our cohort, variation in mild stroke management likely drove a 14.5-percentage-point absolute difference in RT rates despite similar case mix and infrastructure, emphasizing that performance comparisons require careful adjustment for baseline characteristics and subgroup treatment patterns.

Once treatment rates for clearly eligible moderate-to-severe strokes are optimized, variability in treatment of milder cases may have a larger impact on overall RT rates. Given that mild stroke constitutes a large proportion of AIS presentations, variability in this subgroup may substantially influence institutional RT rates and may partly reflect differences in clinical decision-making in the absence of strong selection criteria.

### Comparison with previous studies

Outcome assessment in mild stroke remains challenging, as mRS may lack sensitivity in patients with low baseline disability. Early neurological change assessed by NIHSS is widely used (8, 9), but definitions of improvement vary, and smaller changes may be more appropriate in mild stroke populations.

Evidence regarding RT in mild stroke remains heterogeneous and appears strongly dependent on symptom severity and whether deficits are disabling. Observational studies have reported variable neurological and functional outcomes after thrombolysis (9–16). Randomized evidence in nondisabling minor stroke has not demonstrated a clear functional advantage of thrombolysis over antiplatelet-based treatment, including PRISMS and ARAMIS, while TEMPO-2 similarly found no functional benefit in minor stroke with proven occlusion (17–22). Evidence supporting MT in mild stroke with large-vessel occlusion also remains uncertain (23, 24).

Meta-analyses similarly show limited evidence of functional benefit as measured by mRS, while neurological outcomes assessed using NIHSS are less consistently reported (25–27). Our findings regarding functional outcome are consistent with this evidence, as RT was not independently associated with good functional outcome after adjustment. However, the greater early NIHSS improvement observed among RT-treated patients in unadjusted analyses may reflect the limitations of mRS in mild stroke populations (9).

Recent expert guidance continues to rely on clinician judgment regarding whether symptoms are disabling, reflecting the absence of objective selection criteria (28). This, together with variability in definitions of mild stroke (29), may contribute to inter-center differences in treatment. Because NIHSS may underestimate certain stroke deficits, future refinement of mild stroke definitions may require integration of clinical severity, baseline function, and imaging characteristics rather than reliance on NIHSS and mRS thresholds alone or subjective assessment of symptom severity (16, 23, 30).

Overall, our findings suggest that variability in mild stroke management may reflect differences in treatment thresholds and unmeasured clinical factors rather than infrastructure alone. While higher RT utilization was associated with greater early neurological improvement in descriptive analyses, this did not translate into improved functional outcomes after adjustment, highlighting the need for clearer, consensus-based criteria for RT selection in this population.

### Limitations, strengths and implications

Our study is a real-world comparison of two comprehensive stroke centers operating under the same national framework. Outcome analysis focuses on mild stroke, a large and clinically challenging subgroup of AIS patients that remains under-represented in randomized trials. The inclusion of both short-term neurological and functional outcomes provides complementary perspectives on early recovery in this clinically challenging population.

Limitations include the retrospective design and potential residual confounding. Treatment-center differences likely reflect unmeasured clinical factors and should not be interpreted as causal. Use of two data sources (SITS and hospital databases) may have introduced bias.

Important determinants of RT selection (e.g., vessel occlusion, imaging findings, stroke etiology) were not consistently available for use in statistical analysis. Data on medical management were also lacking. Consequently, although the inter-center variation among patients with mild stroke suggests differences in treatment selection or treatment thresholds, the specific clinical factors responsible for this variation, including the nature and functional impact of neurological deficits and their classification as disabling or non-disabling, cannot be determined from our data. Because NIHSS and mRS were obtained from routine clinical assessments and assessors were not blinded to treatment exposure, detection/measurement bias cannot be excluded.

The relatively small number of RT-treated mild stroke patients may have reduced the statistical power and precision in the multivariable outcome analysis. Accordingly, the absence of an independent association between RT and neurological or functional outcome should not be interpreted as evidence of no treatment effect; a clinically relevant association may have gone undetected.

Outcomes were assessed at hospital discharge. Functional recovery may continue beyond hospitalization, and 90-day mRS is a standard long-term outcome measure in acute stroke studies; however, due to the retrospective design of the study, these data were not consistently available.

Neurological improvement was defined as a ≥ 1-point NIHSS reduction, appropriate for low baseline scores in mild stroke, but potentially influenced by measurement variability. However, a sensitivity analysis using a ≥ 2-point decrease yielded the same overall interpretation. Because fixed mRS thresholds may misclassify outcomes in patients with pre-existing disability, functional outcome was assessed relative to premorbid functional status (31). While this approach aligns with prior studies incorporating baseline function, it may limit comparability with studies using fixed mRS thresholds (31, 32). These limitations should be considered when interpreting the results.

## Conclusion

Inter-center variation in RT utilization between two structurally comparable comprehensive stroke centers was concentrated primarily among patients with mild stroke. Comparable stroke-care infrastructure therefore does not necessarily ensure uniform RT utilization. These findings highlight the need for further studies to better define treatment-selection criteria and understand the clinical determinants of RT use in patients with mild stroke.

## Data Availability

The datasets underlying this article are not publicly available due to ethical and data protection restrictions and cannot be shared beyond the purposes covered by the approved study protocol.
