# Supplementary material for: Beyond the numbers: understanding inter-center variation in recanalization therapy rates
Source: Front Neurol. 2026 Oct 26;17:1898540. doi: 10.3389/fneur.2026.1898540 (PMC13745270; doi:10.3389/fneur.2026.1898540)
Supplement: Supplementary file 1 [file Table_1.docx]

### **SUPPLEMENTARY TABLES**

**Supplementary Table 1.** Baseline characteristics of all acute ischemic stroke (AIS) patients admitted to Tartu University Hospital (TUH) and North Estonia Medical Centre (NEMC) between 1 January and 30 June 2021.

| **Variable** | **TUH**  **n = 332** | **NEMC**  **n = 303** | ***p*-value** |
| --- | --- | --- | --- |
| Age, median (IQR) | 76.0 (66.0–84.0) | 76.0 (66.5–83.0) | 0.62 |
| Onset-to-door time, median (IQR) (h) | 2.8 (1.5–7.6) | 3.4 (1.7–9.3) | 0.430 |
| Early ODT (≤4.5 hr), n (%) | 136 (41.0) | 104 (34.3) | 0.103 |
| Sex: Male, n (%) | 165 (49.7) | 150 (49.5) | 1 |
| NIHSS at admission, median (IQR) | 7.0 (3.0–14.0) | 6.0 (3.0–13.0) | 0.150 |
| NIHSS at discharge, median (IQR) | 3.0 (1.0–9.0) | 3.0 (1.0–7.5) | 0.340 |
| Early arrival among RT recipients, n/N (%) | 113/150 (75.3) | 60/93 (64.5) | 0.043 |
| RT non-recipients’ early arrival, n/N (%) | 23/182 (12.6) | 44/210 (21.0) | 0.142 |
| RT recipients, n (%) | 150 (45.2) | 93 (30.7) | <0.001 |

Values are presented as median (interquartile range) or number (%). Early arrival was defined as onset-to-door time ≤4.5 h and late arrival as >4.5 h. RT = recanalization therapy; AIS = acute ischemic stroke; NIHSS = National Institutes of Health Stroke Scale; TUH = Tartu University Hospital; NEMC = North Estonia Medical Centre.

**Supplementary Table 2.** Baseline characteristics of recanalization therapy (RT) recipients and non-recipients among acute ischemic stroke (AIS) patients by treatment center.

| **Variable** | **RT recipients (n = 243)** | | | **RT non-recipients (n = 392)** | | |
| --- | --- | --- | --- | --- | --- | --- |
|  | **By treatment center** | | | **By treatment center** | | |
|  | **TUH**  **(n =150)** | **NEMC**  **(n = 93)** | **p-value** | **TUH**  **(n = 182)** | **NEMC**  **(n = 210)** | **p-value** |
| Age, median (IQR) | 74.5  (65.0–83.0) | 74.0  (67.0–81.0) | 0.734 | 76.0  (67.0–85.0) | 77.0  (66.0–84.0) | 0.468 |
| Early arrival, n (%) | 113 (75.3) | 60 (64.5) | 0.043 | 23 (12.6) | 44 (21.0) | 0.142 |
| Male sex, n (%) | 77 (51.3) | 45 (48.4) | 0.753 | 88 (48.4) | 105 (50.0) | 0.822 |
| NIHSS at admission, median (IQR) | 8.0  (4.0–15.8) | 12.0 (6.0–19.0) | 0.030 | 4.0  (2.0–12.0) | 4.0  (2.0–8.0) | 0.055 |
| NIHSS at discharge, median (IQR) | 3.0  (0.0–9.0) | 3.0  (0.0–7.0) | 0.853 | 3.0  (1.0–7.0) | 2.0  (1.0–5.0) | 0.674 |

Values are presented as median (interquartile range) or number (%). Early arrival was defined as onset-to-door time ≤4.5 h. AIS = acute ischemic stroke; RT = recanalization therapy; NIHSS = National Institutes of Health Stroke Scale; TUH = Tartu University Hospital; NEMC = North Estonia Medical Centre.

**Supplementary Table 3.** Sensitivity analysis: Multivariable logistic regression analysis of predictors of neurological improvement and good functional outcome among patients with mild acute ischemic stroke (mild stroke) excluding treatment center as a predictor.

| **Predictor** | **NIHSS improvement** | | | **Good functional outcome** | | |
| --- | --- | --- | --- | --- | --- | --- |
|  | **aOR** | **95% CI** | **p-value** | **aOR** | **95% CI** | **p-value** |
| Baseline NIHSS (3–5 vs 0–2) | 4.0 | 2.40–6.57 | <0.001 | 0.6 | 0.33–0.92 | 0.022 |
| Age (per year) | 1.0 | 0.96–1.00 | 0.089 | 1.0 | 0.97–1.01 | 0.283 |
| RT use (yes vs no) | 0.6 | 0.30–1.35 | 0.240 | 0.6 | 0.27–1.11 | 0.096 |
| Sex (male vs female) | 1.0 | 0.59–1.65 | 0.960 | 1.4 | 0.82–2.29 | 0.228 |
| Onset-to-door (>4.5 h vs ≤4.5 h) | 0.8 | 0.40–1.59 | 0.580 | 1.5 | 0.78–2.82 | 0.231 |

Adjusted odds ratios (aOR) with 95% confidence intervals were obtained from multivariable logistic regression including baseline NIHSS category (0–2 vs 3–5), age, sex, onset-to-door time ≤4.5 h, and recanalization therapy use. Neurological improvement was defined as a decrease of ≥1 point in NIHSS between admission and discharge. Good functional outcome was defined as discharge mRS equal to premorbid mRS. TUH = Tartu University Hospital; NEMC = North Estonia Medical Centre; NIHSS = National Institutes of Health Stroke Scale; RT = recanalization therapy.

**Supplementary Table 4**. Sensitivity analysis: Multivariable logistic regression analysis of predictors of neurological improvement defined as a ≥2-point decrease in NIHSS among patients with mild acute ischemic stroke (mild stroke).

| **Predictor** | **aOR** | **95% CI** | **p-value** |
| --- | --- | --- | --- |
| Treatment center (NEMC vs TUH) | 0.55 | 0.30–0.99 | 0.045 |
| Baseline NIHSS (3–5 vs 0–2) | 10.7 | 5.47–20.90 | <0.001 |
| Age (per year) | 0.99 | 0.97–1.01 | 0.363 |
| RT use (yes vs no) | 0.65 | 0.29–1.47 | 0.301 |
| Sex (male vs female) | 1.23 | 0.68–2.21 | 0.495 |
| Onset-to-door (>4.5 h vs ≤4.5 h) | 0.85 | 0.40–1.80 | 0.67 |

Adjusted odds ratios (aOR) with 95% confidence intervals were obtained from multivariable logistic regression including baseline NIHSS category (0–2 vs 3–5), age, sex, onset-to-door time ≤4.5 h, and recanalization therapy use. Neurological improvement was defined as a decrease of ≥2 points in NIHSS between admission and discharge. TUH = Tartu University Hospital; NEMC = North Estonia Medical Centre; NIHSS = National Institutes of Health Stroke Scale; RT = recanalization therapy
